# Hepatotoxicity by Drugs: The Most Common Implicated Agents

**DOI:** 10.3390/ijms17020224

**Published:** 2016-02-06

**Authors:** Einar S. Björnsson

**Affiliations:** Department of Internal Medicine, Division of Gastroenterology and Hepatology, The National University Hospital of Iceland and The Faculty of Medicine, The University of Iceland, 108 Reykjavik, Iceland; einarsb@landspitali.is; Tel.: +354-543-6180; Fax: +354-543-4834

**Keywords:** hepatotoxicity, drugs, drug-induced liver injury, idiosyncratic

## Abstract

Idiosyncratic drug-induced liver injury (DILI) is an underreported and underestimated adverse drug reaction. Information on the documented hepatotoxicity of drugs has recently been made available by a website that can be accessed in the public domain: LiverTox (http://livertox.nlm.nih.gov). According to critical analysis of the hepatotoxicity of drugs in LiverTox, 53% of drugs had at least one case report of convincing reports of liver injury. Only 48 drugs had more than 50 case reports of DILI. Amoxicillin-clavulanate is the most commonly implicated agent leading to DILI in the prospective series. In a recent prospective study, liver injury due to amoxicillin-clavulanate was found to occur in approximately one out of 2300 users. Drugs with the highest risk of DILI in this study were azathioprine and infliximab.

## 1. Introduction

Drug-induced liver injury (DILI) is a frequent differential diagnosis in patients with acute liver injury without obvious etiology. Apart from exclusion of competing etiologies, an important element in the diagnostic process is the information about the known and potential hepatotoxicity of the agent. However, data on hepatotoxicity is not always easily accessible. All drugs approved by regulatory authorities are accompanied by package inserts, called the “patient information” leaflet in Europe and “prescribing information” in the United States [1,2]. Adverse liver reactions are often mentioned in these product labels (package inserts) as a part of the prescribing information. However, it is not always clear whether this is related to enzyme elevations in clinical trials and/or clinically apparent liver injury. Thus, from package inserts of prescribed medications the clinician can get the idea that adverse drug reactions are side effects of most drugs. It has recently been demonstrated that this information is insufficient and even misleading [3]. There was also a substantial discrepancy in the official package inserts and liver disease labeling between Europe and the United States [3]. The documentation of the hepatotoxicity of drugs in the medical literature is very variable.

Some drugs have been convincingly documented to cause liver injury in numerous case reports and case series. Many such drugs have a known clinical signature (phenotype) of liver injury and causality has been further documented by instances of a positive rechallenge [4,5]. Examples are chlorpromazine, halothane, isoniazid and amoxicillin-clavulanate. In early DILI research, halothane and chlorpromazine were commonly reported causes of hepatotoxicity [6]. However, with some drugs, although marketed for many decades, only a single case report or very few reports of liver injury have been published. Case reports are often not well described and critical clinical information is frequently lacking [7]. A recent study found that reports of drug-induced liver diseases often did not provide the data needed to determine the causes of suspected adverse effects [7]. Although a case report has been published, it does not prove that the drug is hepatotoxic.

A newly established website, LiverTox^®^ [8], was an attempt to provide up-to-date, accurate, and easily accessible information on the diagnosis, causes, frequency and patterns of liver injury attributable to both prescription and nonprescription medications. In LiverTox^®^ there is data on almost all medications marketed in the United States, both on those who have been reported to cause liver injury and those without reports of liver injury. Although in LiverTox^®^ a thorough literature search has been undertaken and is provided, no attempt has been made to judge the quality of the published reports or the causality of the suspected liver injury reported.

In a recently published paper, drugs in LiverTox^®^ were classified into categories, using all reports in this website [9]. For drugs with rather few reports (<12), the Rousel Uclaf Causality Assessment Method (RUCAM) was used [10]. In this critical analysis, many of the published reports did not stand up to critical review and currently there is no convincing evidence for some drugs with reported hepatotoxicity to be hepatotoxic [9]. Although certain drugs have a distinct phenotype such as isoniazid, which generally leads to a hepatocellular pattern or chlorpromazine cholestatic liver damage, many drugs can lead to both hepatocellular and cholestatic injury. Listing all types of patterns that have been reported for all these drugs is unfortunately not possible in this paper.

## 2. Categories of Hepatotoxicity

In the creation of LiverTox, drugs were arbitrarily divided into four different categories of likelihood for causing liver injury based on reports in the published literature [8]. Category A with >50 published reports, B with >12 but less than 50, C with >4 but less than 12, and D with one to three cases. In the Hepatology paper, drugs were categorized based on these numbers and another category, T, was added for agents leading to hepatotoxicity mainly in higher-than-therapeutic doses [9]. The number of published cases was counted unless >100 cases were found. The analysis was based mainly on published case reports, but case series were used if a formal causality assessment had been undertaken.

In the analysis of the hepatotoxicity of drugs found in LiverTox, fewer drugs than expected had documented hepatotoxicity. Among 671 drugs available for analysis, 353 (53%) had published convincing case reports of hepatotoxicity. Thus, overall, 47% of the drugs listed in LiverTox did not have evidence of hepatotoxicity. This is at odds with product labeling which very frequently lists liver injury as adverse reaction to drugs [3]. It has to be taken into consideration that 116/863 (13%) of marketed agents had be excluded from the analysis. New drugs approved within the last five years were not included as most instances of hepatotoxicity appear in the post-marketing phase [11]. Metals (iron, nickel, arsenic), illegal substances (cocaine, opium, heroin), and infrequently used and/or not available (not marketed currently) drugs were also excluded [9]. Herbal and dietary supplements listed in LiverTox were not included in the category analysis.

Among the 671 drugs available for analysis, the proportions of the drugs in the different categories were: A, 48 (14%); B, 76 (22%); C, 96 (27%); and D, 126 (36%). A total of 318 (47%) drugs have not been implicated (category E).

In general, drugs in categories A and B were more likely than those in C and D to have been marketed for a long time, and both were more likely to have at least one fatal case of liver injury and reported cases of positive rechallenge. There is little doubt that drugs with >50 or 100 published reports of DILI such as category A drugs are hepatotoxic. The same is probably true for the vast majority of drugs in category B. However, in categories C and D with one to 12 cases reported, it is still not clear whether these agents are really hepatoxic drugs.

## 3. Category A

Although drugs in this category (*n* = 48) were supposed to have >50 case reports of liver injury associated with the use of these drugs, 81% of the drugs had >100 cases reported. Interestingly, overall, 92% of these drugs had documented positive rechallenge. In Table 1, the category A drugs are illustrated with the indication and/or class of drug. These agents in category A are the real potential hepatotoxins and clinicians should be aware of that when evaluating the risk-benefit ratio of drug therapy. Treatment with these drugs should motivate physicians to guide patients about potential symptoms of liver injury when taking these drugs and about prompt discontinuation if these symptoms occur. All except one entity (estrogens-progestins) or 98% had at least one convincing case that was associated with fatal outcome. All of these drugs except telithromycin had been approved for marketing for more than 15 years and 63% for more than 35 years [9]. The most common types of drugs were antimicrobials among 33% of the drugs, followed by drugs acting on the central nervous system (12.5%), cardiovascular (12.5%), rheumatologic (12.5%), antineoplastic (10%), endocrine (6%) and other types of drugs (13%). Although antimicrobials were the most common agents among drugs, antimicrobials were also the most common agents in categories B (30%), C (19%) and D (27%). Antibiotics have been shown to be the dominating type of drug in both prospective and retrospective studies on DILI [12,13,14,15,16]. There is unfortunately not enough room to discuss many of these well-documented hepatotoxic agents. As mentioned in the abstract, azathioprine and infliximab have in one study been found to be associated with the highest risk of liver injury [9]. Both hepatocellular and cholestatic injury has been described due to azathioprine [8,9]. Despite the common problem of hepatotoxicity with azathioprine, there is a lack of studies with a significant number of well-characterized patients with this type of liver injury.

**Table 1:** Drugs that, according to analysis of data in LiverTox [8], have been associated with more than 100 cases of drug-induced liver injury.

Drug	Drug Class/Indication
1. Allopurinol	Gout prophylaxis
2. Amiodarone	Arrhythmia
3. Amoxicillin-clavulanate	Antibiotic
4. Anabolic steroids	Body building
5. Atorvastatin	Lipid lowering agent
6. Azathioprine/6-Mercaptopurine	Immunosuppressive agent
7. Busulfan	Malignancy
8. Carbamazepine	Antiepileptic
9. Chlorpromazine	Psychosis
10. Contraceptives	Birth control
11. Dantrolene	Muscle relaxant
12. Diclofenac	NSAID
13. Didanosine	Antimicrobial
14. Disulfiram	Substance abuse agent
15. Efavirenz	Antimicrobial
16. Erythromycin	Antimicrobial
17. Floxuridine	Antineoplastic
18. Flucloxacillin	Antimicrobial
19. Flutamide	Antineoplastic
20. Gold salts	Immunosuppressive agent
21. Halothane	Anaesthetic
22. Hydralazine	Antihypertensive
23. Ibuprofen	NSAID
24. Infliximab	Immunosuppressive agent
25. Interferon alpha/Peginterferon	Antimicrobial
26. Interferon beta	Multiple Sclerosis
27. Isoniazid	Antituberculosis
28. Ketoconazole	Antifungal
29. Methotrexate	Immunosuppressive agent
30. Methyldopa	Antihypertensive
31. Minocycline	Antibiotic
32. Nevirapine	Antimicrobial
33. Nimesulide	NSAID
34. Nitrofurantoin	Antibiotic
35. Phenytoin	Antiepileptic
36. Propylthiouracil	Antithyroid
37. Quinidine	Arrhythmia
38. Pyrazinamide	Antituberculosis
39. Rifampin	Antituberculosis
40. Simvastatin	Lipid lowering agent
41. Sulfamethoxazole/Trimethoprim	Antibiotic
42. Sulfazalazine	Antibiotic
43. Sulfonamides	Antibiotic
44. Sulindac	NSAID
45. Telithromycin	Antibiotic
46. Thioguanine	Antineoplastic
47. Ticlopidine	Platelet inhibitor
48. Valproate	Antiepilepitic

## 4. Category B

As mentioned above, most of these drugs with >12 and up to 50 case reports of DILI published probably carry hepatotoxic potential. This seems particularly true for drugs with reports of documented rechallenge, which had been reported in at least one case in 38% of the drugs [9]. In comparison with category A drugs, which almost exclusively had been associated with fatality, approximately 50% of category B drugs had been associated with a fatal outcome. Thus, in drugs with less frequent reporting of liver injury in category B, only 38% had rechallenge reported *vs.* 92% in category A, which suggests that the “proof” of hepatotoxicity is not there for all these drugs. In category B, 13/76 (17%) drugs with >30 cases reported are shown in Table 2.

**Table 2:** Drugs in category B (>12 and >40 cases) that, according to analysis of data in LiverTox [8], have been associated with >30 published case reports of drug induced liver injury.

Drug	Drug Class/Indication
Amodiaquine	Antimicrobial
Azithromycin	Antimicrobial
Chlorzoxazone	Muscle relaxant
Cyproterone	Antineoplastic
Heparin	Anticoagulant
Imatinib	Antineoplastic
Irinotecan	Antineoplastic
Levofloxacin/Ofloxacin	Antimicrobial
Oxacillin	Antimicrobial
Phenobarbital	Antiepileptic
Stavudine	Antimicrobial
Tamoxifen	Antineoplastic
Terbinafine	HIV

## 5. Categories C, D and E

Overall, 222/353 (63%) of drugs in LiverTox^®^ with hepatotoxicity fall into categories C and D. Compared with category D, with only one to three cases reported, category C (<12 and >4 case reports) drugs were more likely to have rechallenge reports, with 26% *vs.* 11%, and fatal cases of 23% and 7%, respectively. A positive rechallenge is usually defined with biochemical criteria, showing recurrence of liver test abnormalities upon readministration of the drug, due to either intentional or inadvertent re-exposure [4,5]. This is generally considered to be the gold standard of the diagnosis of drug-induced liver injury. A documented positive rechallenge provides more evidence of the hepatotoxicity of a given drug. Given the frequency of case reports with drugs in categories A and B, there seems little doubt that drugs in these categories can lead to hepatotoxicity and little need to do a strict causality assessment of reports with these drugs.

However, in category C, consisting of 4–11 case reports, the hepatotoxicity of some drugs can be put into question. To illustrate this, 16 drugs in this category only had case reports with a possible likelihood score according to RUCAM. None of these drugs had documented fatal liver reactions or rechallenge. Thus, it can be concluded that these drugs do not have a well-documented hepatotoxicity, although liver injury with their use cannot be excluded. The poorly documented exclusion of competing causes, as well as the use of other concomitant drugs, made a causality assessment difficult. This has been problematic in many reports of suspected hepatotoxicity with human immunodeficiency virus (HIV) drugs [17,18,19]. It is very important that observations of hepatotoxicity of new drugs should lead to well-documented case reports with detailed clinical and biochemical information.

The analysis reported in the Hepatology paper revealed that many drugs labeled as hepatotoxic and with a single or few case reports suggesting hepatotoxicity did not fulfill causality criteria by use of the RUCAM instrument [9].

## 6. Common Drugs Leading to Liver Injury in Drug-Induced Liver Injury (DILI) Studies

As mentioned above, antibiotics have, in all prospective studies, been found to be the most common drugs leading to hepatotoxicity [12,13,14,15,16]. In the most recently published series from the DILIN cohort in the US, antimicrobials, including antibacterial agents and antituberculosis agents, were approximately 46% of all DILI cases [20]. Furthermore, among the top 10 drugs in the DILIN registry, all drugs except one (Diclofenac) are antibiotics [20]. Table 3 illustrates the five most common drugs associated with liver injury in at least three prospective studies. Interestingly, all of these drugs belong to category A.

**Table 3:** The top five implicated drugs in three prospective studies on DILI, in Spain (Andrade *et al.* [12] 2005), liver injury in drug-induced liver Injury (DILI) study from the US (Chalasani *et al.* [13] 2013) and a prospective study from Iceland (Bjornsson *et al.* [14] 2015).

Spanish Registry	DILIN Study	Icelandic Study
Amoxicillin-clavulanate	Amoxicillin-clavulanate	Amoxicillin-clavulanate
Isoniazid	Isoniazid	Diclofenac
RIP + INH + PIZ	Nitrofurantoin	Azathioprine
Flutamide	SMZ/TMP	Infliximab
Ibuprofen	Minocycline	Nitrofurantoin

RIP + INH + PIZ: Rifampin, Isoniazid and Pyrazinamide; SMZ/TMPSulfamethoxazole/Trimethoprim.

In India, anti-tuberculous drugs (58%), anti-epileptics (11%), olanzapine (5%), and dapsone (5%) were the most common causes [16]. A unified list of drugs associated with DILI was recently established [21]. Overall 385 individual drugs were identified; 319 drugs were identified in three DILI registries, *i.e.*, from Spain, Sweden and the US. The 10 most frequently implicated drugs were: amoxicillin-clavulanate, flucloxacillin, erythromycin, diclofenac, sulfamethoxazole/Trimethoprim, isoniazid, disulfiram, Ibuprofen and flutamide [12,13,14,21].

## 7. Risk of DILI among Patients Using Potentially Hepatotoxic Drugs

Previously, data on numbers needed to harm drug users in terms of liver injury has been limited. Several retrospective case control cohort studies using the General Practitioners Research Database (GPRD) were the first studies on this [22,23,24].

A risk of DILI greater than 100 per 100,000 users was found for chlorpromazine and isoniazid. Drugs with an intermediate risk were amoxicillin-clavulanic acid and cimetidine, with a risk of one per 10 per 100,000 users [24]. All other drugs were found to be less than 10 per 100,000 users. The following drugs were most strongly associated with DILI: Chlorpromazine, amoxicillin-clavulanic acid, flucloxacillin, macrolides, tetracyclines, metoclopramide, chlorpheniramine, betahistine, sulfasalazine, azathioprine, diclofenac, and antiepileptics The highest crude incidence rates were one per 739 users (chlorpromazine), one per 1103 (azathioprine), one per 1000 (sulfasalazine), and one per 11,688 (amoxicillin-clavulanate). The limitations of this study were the retrospective design with a lack of complete data regarding diagnostic testing and a lack of data on over-the-counter drugs and herbal agents [24]. In a recent prospective study on DILI from Iceland, data on the use of drugs was available [9]. The risk of DILI among patients using potentially hepatotoxic drugs could therefore be calculated. Amoxicillin-clavulanate-induced liver injury was found in one of 2350 outpatient users, which was higher among those who were hospitalized already, one of 729. This might be due to a detection bias, with more routine testing of the liver in the hospital, but it cannot be excluded that sicker patients are more susceptible to liver injury from this drug. The incidence rates were higher than previously reported, with the highest being one of 133 users for azathioprine and one of 148 for infliximab.
